# A stem cell-derived ovarian regenerative patch restores ovarian function and rescues fertility in rats with primary ovarian insufficiency

**DOI:** 10.7150/thno.61690

**Published:** 2021-08-18

**Authors:** Sichen Zhang, Dashuai Zhu, Zhenhua Li, Ke Huang, Shiqi Hu, Halle Lutz, Mengjie Xie, Xuan Mei, Junlang Li, Genevieve Neal-Perry, Shaowei Wang, Ke Cheng

**Affiliations:** 1 Department of Gynecology and Obstetrics, Beijing Hospital, National Center of Gerontology; Institute of Geriatric Medicine, Chinese Academy of Medical Sciences, P.R. China. NO.1 DaHua Road, Dong Dan, Beijing 100730, P. R. China.; 2 Joint Department of Biomedical Engineering, University of North Carolina at Chapel Hill and North Carolina State University, Raleigh, NC 27695, USA.; 3 Molecular Pharmaceutics Division, Eshelman School of Pharmacy, University of North Carolina at Chapel Hill, Chapel Hill, NC 27599, USA.; 4 Peking Union Medical College, Chinese Academy of Medical Sciences, Graduate School of Peking Union Medical College. No. 9 Dong Dan Santiao, Beijing 100730, P.R. China.; 5 Department of Obstetrics and Gynecology, School of Medicine, University of North Carolina at Chapel Hill, Chapel Hill, NC 27599, USA.; 6 Division of Pharmacoengineering and Molecular Pharmaceutics, Eshelman School of Pharmacy, University of North Carolina at Chapel Hill, Chapel Hill, NC 27599, USA.

**Keywords:** primary ovarian insufficiency, ovarian regenerative patch, regenerative medicine, mesenchymal stem cells, acellular therapy, fertility

## Abstract

**Rationale:** Primary ovarian insufficiency (POI) normally occurs before age 40 and is associated with infertility. Hormone replacement therapy is often prescribed to treat vasomotor symptom, but it cannot restore ovarian function or fertility. Stem cell therapy has been studied for the treatment of POI. However, the application of live stem cells has suffered from drawbacks, such as low cell retention/engraftment rate, risks for tumorigenicity and immunogenicity, and lack of off-the-shelf feasibility.

**Methods:** We developed a therapeutic ovarian regenerative patch (ORP) that composed of clinically relevant hydrolysable scaffolds and synthetic mesenchymal stem cells (synMSCs), which are microparticles encapsulating the secretome from MSCs. The therapeutic potency of ORP was tested in rats with cisplatin induced POI injury.

**Results:***In vitro* studies revealed that ORP stimulated proliferation of ovarian somatic cells (OSCs) and inhibited apoptosis under injury stress. In a rat model of POI, implantation of ORP rescued fertility by restoring sexual hormone secretion, estrus cycle duration, and follicle development.

**Conclusion:** ORP represents a cell-free, off-the-shelf, and clinically feasible treatment for POI.

## Introduction

The prevalence of primary ovarian insufficiency (POI) is as high as 3.7% in worldwide and is defined by the deficiency of ovarian function before 40 1, 2. It is characterized by amenorrhea (> 6 months) in the setting of elevated follicle-stimulating hormone (FSH) level and a hypo- or eugonadism 3. Women affected by POI fail to undergo the pubertal transition and suffer from vasomotor symptoms, sleep disturbance, genitourinary syndrome, reduced skin elasticity, emotional irritability, increased risks for cardiovascular disease, and infertility 4. Current strategies for POI treatment include improving life qualities, such as adopting healthy lifestyle behaviors, hormone replacement therapy (HRT) and puberty induction 5. However, these treatments fail to reverse ovarian functions, such as female sex hormone levels, follicular development, or ovulation 6. Therefore, efficient strategies designed to restore ovarian physiology would benefit POI-affected patients 7.

**Figure 1:**
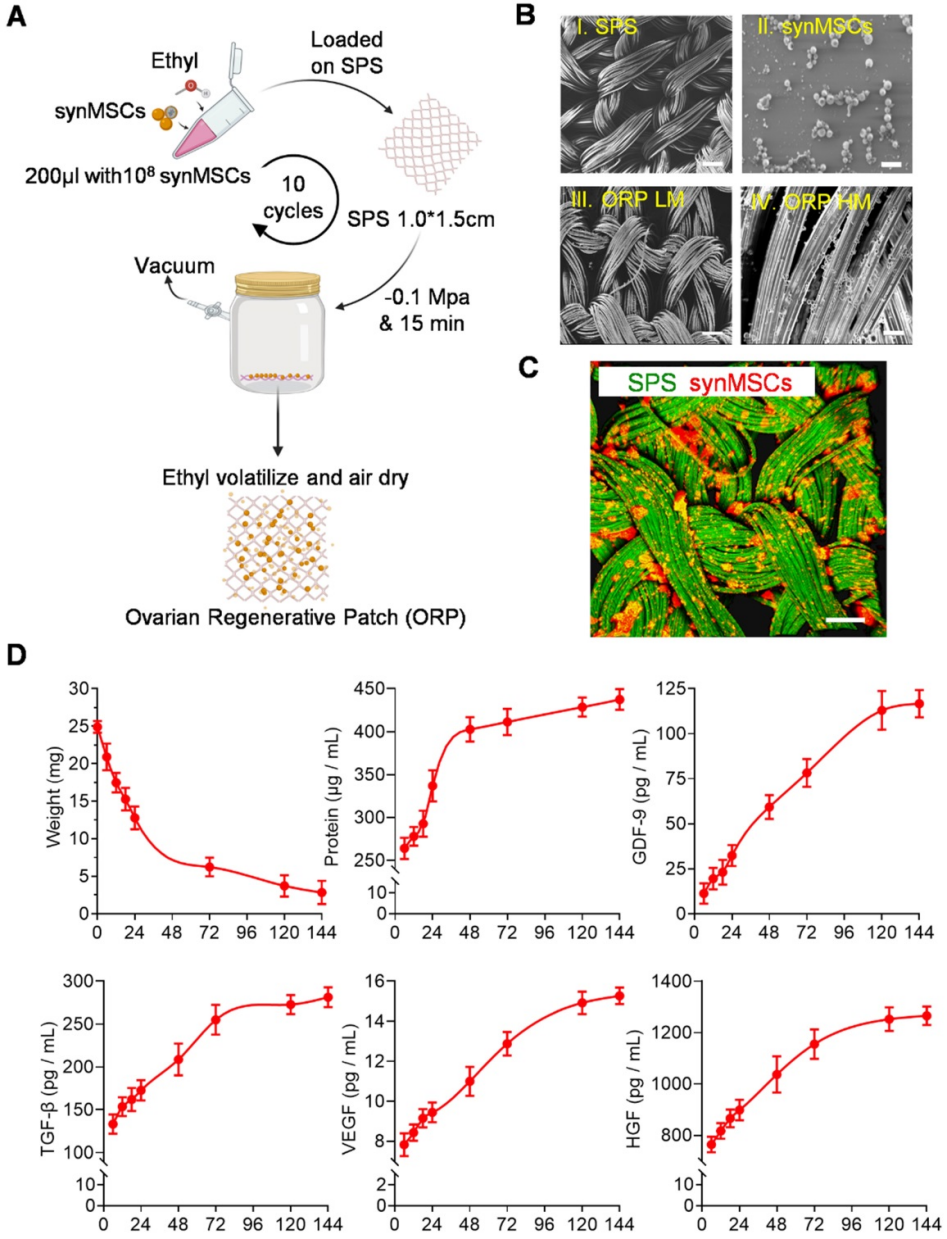
** Fabrication and characterization of ORP. (A)** Schematic showing the method of ORP production. **(B)** Representative SEM images showing the cross-sectional view of I) SPS, Scale bar: 100 µm. II) synMSCs, Scale bar: 5 µm. III) ORP low magnification, Scale bar: 10 µm. IV) ORP high magnification, Scale bar: 5 µm. **(C)** Representative fluorescence micrographs showing ORP composed of SPS (green) and synMSCs (red), Scale bar: 50 µm. **(D)** Quantitative analysis of ORP weight (*n* = 3), the cumulative release of total protein, growth differentiation factor-9 (GDF-9), transforming growth factor-β (TGF-β), vascular endothelial growth factor (VEGF), and hepatocyte growth factor (HGF) from ORP were analyzed by ELISA (*n* = 3). All data are presented as mean ± SD. SPS, surgical pelvic scaffold, synMSC, synthetic mesenchymal stem cell, ORP LM, ovarian regenerative patch low magnification, ORP HM, ovarian regenerative patch high magnification.

Stem cell therapy holds great potentials for regenerative treatment of POI 8-10. Abundant evidence in preclinical 11, 12 and clinical 13 research indicated that MSCs repair injured ovaries through a paracrine mechanism. MSCs had been established as a progressing therapeutic for POI treatment by releasing paracrine of regenerative factors 12, 14, 15. However, the clinical application of stem cell therapy has been hampered by many intrinsic limitations including the risk of immunogenicity and tumorigenicity 16, 17. Stem cell-derived secretome has been studied as an alternative to stem cells 18, 19. The challenge is how to deliver secretome to ovaries with effective retention and minimal invasiveness 20. To solve those problems, we designed an ovarian regenerative patch (ORP) by embedding synthetic mesenchymal stem cells (synMSCs) into a clinically approved surgical pelvic scaffold (SPS) (Figure 1A). After that, we evaluated the physicochemical mechanical and biological properties for ORP both *in vitro* and *in vivo*. Additionally, risks of ORP implantation, therapeutic efficacy and long-term outcomes were assessed in an established rat model of POI 21, 22.

## Methods

### Design of study

Our research aimed to produce an off-the-shelf ORP to rescue ovarian function in POI and overcome barriers that have prevented the use of living stem cells to treat POI (Figure S1). ORPs were fabricated by embedded Polylactic glycolic acid (PLGA) which is encapsulated secretomes derived from MSCs (synMSCs) into a decellularized surgical pelvic scaffold (SPS). We characterized the 3D structures, releasing properties, and therapeutic potency of ORP. *In vivo*, a rat POI model was used to explore the distribution, biocompatibility, safety, efficacy and potential mechanism of ORP therapy. Animals were set in control or treatment groups randomly. Ovarian function, fertility and histology were evaluated for therapeutic effects. Animal work was allowed by the Institutional Animal Care and Use Committee at the North Carolina State University.

### Preparation of synMSCs

Human umbilical cord MSCs (Huc-MSCs) (Cyagen, CA, USA) were cultured as previously described 30. Briefly, the Huc-MSCs were cultured with 10% fetal bovine serum (FBS) media in incubator with an environment of 5% CO_2_ and 37 °C. When the density of cells was 80-90%, we washed cells by changing FBS-free media every 30 mins with PBS raise. Three days later, the conditioned media was collected, then cell debris and contaminants were removed by filtering through a 0.22 μm filter. Next, the sterilized conditioned media was stored at -80 °C for 24 hours before lyophilization (LABCONCO FreeZone 2.5 Liter Freeze Dry System) for purifing Huc-MSCs secretomes. To fabricate the synMSCs, we used PLGA as a nontoxic building block for sustained release. SynMSC was fabricated by a protocol of double emulsion followed by membrane extrusion 20. Briefly, Huc-MSCs secretomes as the internal aqueous phase in 0.1% poly-vinyl alcohol (PVA) were injected into dichloromethane (DCM) contained PLGA as oil phase. Then, the whole content was sonicated for seconds under a 4 °C environment. Then, the emulsion was injected into solution of 0.7% PVA immediately. The secondary emulsion was emulsified for 12 min on a high-speed homogenizer for the final water/oil/water (w/o/w) mixture. Then, to promote solvent evaporation, magnetic bar stirred it continuously overnight. For microscopic examination, we used PLGA-Rhodamine B (Nanosoft Polymers, NSP, NC, USA). Solidified synMSCs were stored at -80 °C until use. The diameters of synMSCs were 5-10 μm.

### Fabrication and characterization of ORP

SynMSCs were fabricated by encapsulating secretomes derived from Huc-MSCs into biodegradable PLGA microparticles 23, 24. SynMSC achieves its therapeutic function via mimicking the paracrine activities of natural cells 23. The surgical pelvic scaffold 25 was reported as oxidized regenerated cellulose that can safely reduce the clinical relevant consequences of local wound bleeding and tissue adhesions 26. To prepare the ORP, the synMSCs were suspended in ethyl instead of phosphorylated buffer saline (PBS) or water to avoid SPS (Gynecare Interceed Adhesion Barrier, Johnson and Johnson) hydrolyzation during embedding procedure. Then, the synMSCs suspension (200 μL, 5×10^8^/ml) was added on the SPS (1.0 × 1.5 cm) and immediately placed in vacuum chamber until ethyl completely evaporated (10 repeated cycles per SPS). We labeled scaffolds with fluorescein isothiocyanate isomer (FITC) (Sigma, MO, U.S.) and prelabeled synMSCs with Rhodamine B for microscopic visualization. Images were taken from the fluorescence microscope (Olympus IX81).

### Scanning Electron Microscope (SEM)

The morphology of the ORP was visualized via scanning electron microscopy (SEM). Briefly, samples were coated with a thin golden layer and mounted on an aluminum stub. Then, samples were scanned and imaged under a scanning electron microscope (JEOL 6010LA SEM, JEOL Ltd., Japan).

### ORP Releasing experiment

We measured the weight of ORP with an electronic scale after dehydration at each time point (0, 6, 12, 18, 24, 72, 120, 144h). The total protein concentration was measured by protein quantitation assays (Thermo Scientific, MA, USA). Growth factors including GDF-9 (Detection range: 2.2 ng/mL - 500 ng/mL, Raybiotech Inc, GA, U.S.), VEGF (Assay range: 0.32 pg/mL -25000 pg/mL, Thermo Scientific, MA, U.S.), TGF-β (Assay range: 31 pg/mL -2,000 pg/mL, Thermo Scientific, MA, U.S.), and HGF (Assay range: 63 pg/mL -4,000 pg/mL, Thermo Scientific, MA, U.S.) were detected by ELISA kit.

### Isolation of ovarian somatic cells

Ovaries from rats were placed in cold, sterile PBS containing 1% gentamicin immediately after removing from the animals. Then, ovaries were cut into small pieces and placed in 2% collagenase IV for an hour to dissociate. Next, the same volume of medium contained 20% FBS was used to terminate the dissociation. After centrifuge, the supernatant was removed and the pellets were resuspended with a medium containing 10% FBS. The cells were transferred to T-175 flasks and culture at 37 °C incubators, with 5% CO_2_. The media was replaced every 24 hours, and after five days, the cells were subjected to flow cytometry for characterization of ovarian somatic cells (OSCs).

### Rat model of POI

Animal care and experimental protocols were done in accordance with the Institutional Animal Care and Use Committee (IACUC) of North Carolina State University. Cisplatin is a class of alkylating agents that cross-linking bases in DNA strands and results of cell death via disruption of DNA function 27. POI was induced in 5-7 week female Sprague-Dawley rats (Charles River Laboratories) by injecting cisplatin (2.5 mg/kg/d, 22-515-0, Fisher, USA) for seven consecutive days 28. Control rats were injected with saline. Estrous cyclicity was used to monitor ovarian function and to confirm the establishment of rat POI model.

### Examination of estrous cycles

Vaginal smears were used to determine estrous cycling. Briefly, rats were subjected to vaginal lavage for five consecutive days. We defined the estrous cycle as previously reported 29. Proestrus is defined as equal leukocytes and elongated nuclear epithelium. Estrus is defined as most large cornified epithelial cells; Metestrus is defined as similar number of leukocytes, elongated nuclear epithelium and large cornified epithelial cells. Diestrus is defined as leukocytes mostly.

### ELISA for rat hormone

Tail vein blood was collected from the rats at baseline and three weeks after treatments. Serum was separated using centrifugation. The concentrations of serum FSH (Assay range: 1 ng/mL- 25 ng/mL, 89-100-589, Fisher, USA), E2 (Assay range: 20 pg/ml - 2000 pg/ml, ab108667, Abcam, USA) and AMH (Assay range: 0.164 ng/ml - 40 ng/ml, ab267629, Abcam, USA) were measured with ELISA kits.

### Ovary morphometry and follicle examination

Ovaries were harvested and cut into 5 μm sections, and every fifth section was used for assessing ovarian histology after H&E staining. A Path Scan Enabler IV slide scanner (Advanced Imaging Concepts, USA) took low power field images. The total account of follicles in each section was counted and the percentage of all types of follicles was calculated respectively. The categories of follicles can be defined as primordial when it possessed an oocyte around a single layer of flat squamous granulosa cells; primary when it possessed an oocyte surrounded by a layer of cuboidal granulosa cells; secondary when it possessed an oocyte surrounded by more than two layers of granulosa cells, and atretic when a single large space showed. TEM scanning was performed by the Analytical Instrumentation Facility (AIF) of North Carolina State University. After collecting, the samples were fixed in a mixture of 4% paraformaldehyde and 1% glutaraldehyde, followed by embedding and sectioning, the TEM images were acquired.

### Flow cytometry

To characterize the phenotype of Huc-MSCs, OSCs and OSCs apoptosis, flow cytometry performing undergoes a Beckman Coulter flow cytometer (Brea, CA). Huc-MSCs were characterized by primary antibodies, including CD29 (ab30394), CD31 (ab119339), CD105 (ab107595), CD34 (ab81289), CD90 (ab226), and CD45 (ab10558). All antibodies were acquired from Abcam. FSHR (22665-1-AP, Thermo Fisher Scientific) was used for characterization of OSCs. ANNEXIN V-FITC apoptosis kit (K101-100, Biovision) was used for apoptosis assay by referring to the manufacturer manual (V13242, Thermo Fisher).

### Immunohistochemistry (IHC)

Ovarian cryo-sections were fixed by paraformaldehyde (PFA) solution, then blocked with blocking solution that contains 0.01% saponin (Sigma-Aldrich). Primary antibodies of FHSR (1:200, 22665-1-AP, Thermo Fisher), Ki67 (1:200; 14-5698-82, invitrogen), and CD31 (1:200, ab119339, abcam) diluted in protein a blocking solution were incubated with the sections overnight at 4 °C. After washing with PBS, Alexa Fluoro-488 and Alexa Fluoro-594 conjugated secondary antibodies were incubated with the samples for 2 hours at room temperature. Nuclei were counterstained using Prolong Gold with DAPI (P36935, Thermo Fisher). TUNEL assay was performed using a commercial kit (G3250, Promega). Fluorescent images were captured from a confocal laser scanning microscope (Olympus, FV3000). Bright field images were acquired by using Echo Revolve microscope (ECHO, United States).

### Statistical analysis

Statistical analysis was performed using GraphPad Prism software (Version 9.0.1, GraphPad Software, United States). Data were expressed as mean ± SD, at least three independent tests were performed in each study, and similar results were acquired. Comparison between two groups was performed with a two-tailed, unpaired Student's t test. One way-ANOVA was used for comparison of more than three groups followed with Bonferroni test. Differences were considered statistically significant when *P* < 0.05.

## Results

### Fabrication and characterization of ORP

Consistent with previous reports 29, 31, 32, Huc-MSC purification was achieved and verified by immunocytochemistry (ICC) and flow cytometry of cell biomarker expression (Figure S2A and B). SynMSCs were fabricated using PLGA to encapsulate the secretome derived from Huc-MSCs as described in our previous work 16. Here, we embedded synMSCs in the hydrolysable SPS via ethyl volatilization method (Figure 1A). The count of synMSCs on one ORP was more than 1000 million. Scanning electron microscope (SEM) demonstrated that SPS structure change was neglectable before and after synMSC embedding (Figure 1B). synMSCs were found present throughout the depth of the SPS (Figure 1C, S3, and Video S1). Weight change analysis of ORP indicated that it was hydrolyzed in six days (Figure 1D). The release function ORP was evaluated by measuring the cumulative release of total protein and growth factors, including growth differentiation factor-9 (GDF-9), transforming growth factor-β (TGF-β), vascular endothelial growth factor (VEGF), and hepatocyte growth factor (HGF) (Figure 1D). We found an initial increase followed by a sustained release of each hormone for more than 144 hours. These results suggested that ORP achieved sustainable growth factor release plateau along with structure hydrolyzation in about six days.

### Regenerative effects of ORP *in vitro*

**Figure 2:**
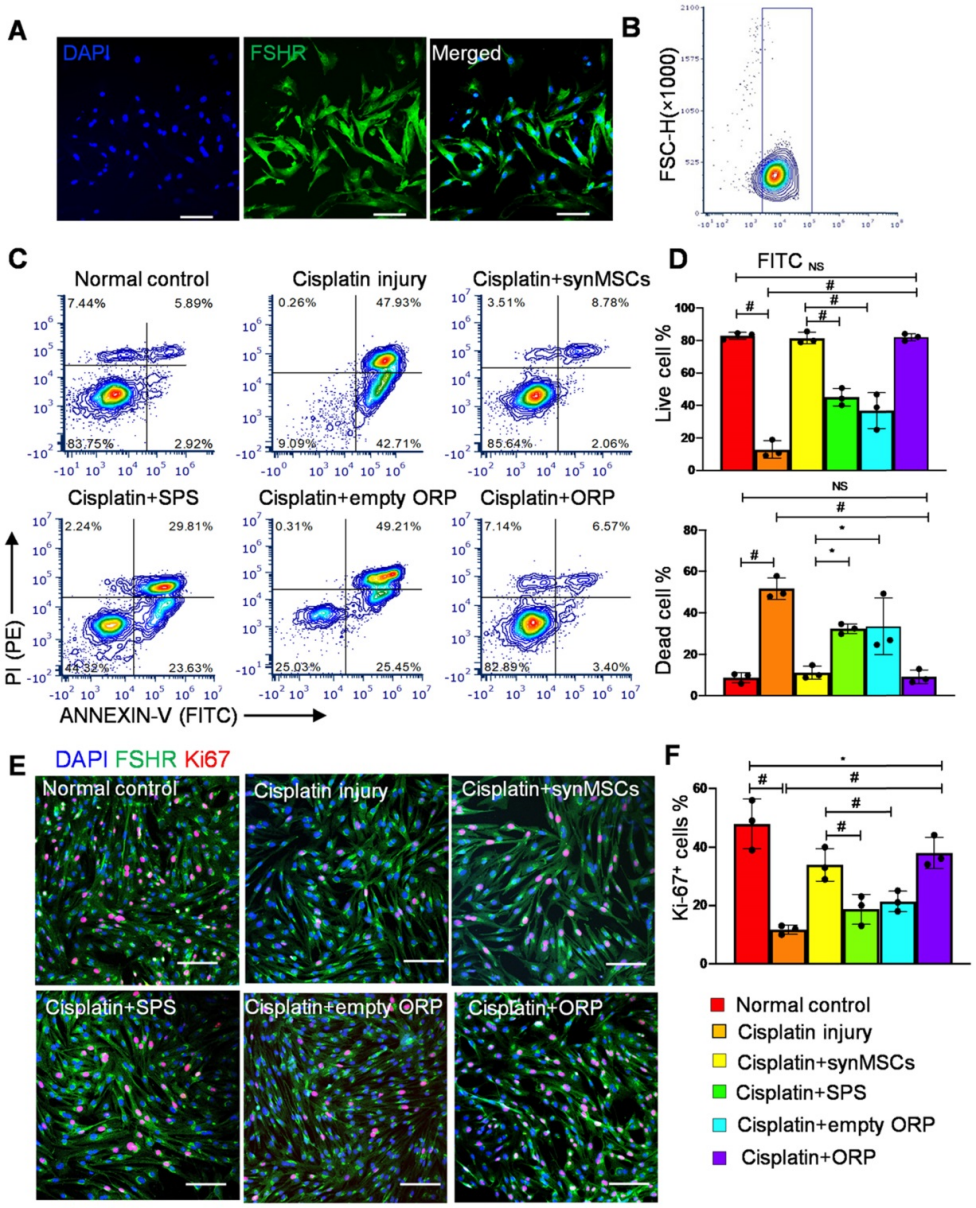
** Regenerative effects of ORP *in vitro*. (A)** Representative immunocytochemistry (ICC) fluorescence micrographs showing FSHR expression (green) in isolated cells. Scale bar: 100 µm. **(B)** Flow cytometry analysis of FSHR expression in isolated cells. **(C)** Flow cytometry of apoptotic OSCs stained with ANNEXIN V-FITC and PI-PE kit (*n* = 3). **(D)** Quantitative analysis of live cells and dead cells in control, ciOSCs, ciOSC treated with synMSCs, SPS, empty ORP, or ORP via flow cytometry (*n* = 3). **(E)** ICC fluorescence images showing Ki67^+^ expression (red) in different groups of FSHR^+^ OSCs (green), including normal control, cisplatin injury control, injured OSCs treated with synMSCs, SPS, empty ORP, or ORP. Scale bar: 50 µm (*n* = 3). **(F)** Quantitative analysis of Ki67^+^ cells in normal control, ciOSCs and ciOSCs treated with synMSCs, SPS, empty ORP, or ORP. (*n* = 3). All data are presented as mean ± SD. Student's t-test for comparison between two groups and one-way ANOVA for comparison among two and more groups. * indicates *P* < 0.05; ** indicates *P* < 0.01; ^#^ indicates *P* < 0.0001.

OSCs were isolated from ovarian follicles in adult female Sprague Dawley (SD) rats 33, 34. Follicle-stimulating hormone receptor (FSHR) is a transmembrane marker on OSCs 35. Immunocytochemistry (ICC) staining of FSHR confirmed the identity of OSCs (Figure 2A). Flow cytometry of FSHR showed that the purity of isolated OSCs was above 90% (Figure 2B). In this study, we treated OSCs with cisplatin to establish an *in vitro* model of POI 35. OSC injury was confirmed by flow cytometry on ANNEXIN-V-FITC/PI apoptosis detection (Figure 2C, D). To test the therapeutic potency of ORP *in vitro*, cisplatin injured OSCs (ciOSCs) were treated with ORP for 24 hours and then analyzed via ANNEXIN-V-FITC/PI staining and FACS flow cytometry, and ICC staining of Ki67, a marker of cell proliferation (Figure 2C-F). The percentage of live, dead cells and Ki67 positive cells were quantified respectively. Compared to the control group, both ORP and synMSCs treatment significantly enlarged the proportion of live OSCs and reduced apoptotic OSCs proportion (Figure 2C, D). Treatment with SPS or empty ORP did not have such effects (Figure 2C, D). The number of Ki67 positive proliferating cells was also increased by ORP treatment (Figure 2E, F). Treatment with synMSCs had similar results. These results showed that treatment with ORP protects OSCs from cisplatin-induced apoptosis. Similar results were obtained using ovarian epithelial cells (Figure S4). Taken together, ORP effectively improved proliferation and reduced apoptosis in ovarian granulosa and epithelial cells.

### Successful establishment of a rat model of POI

**Figure 3:**
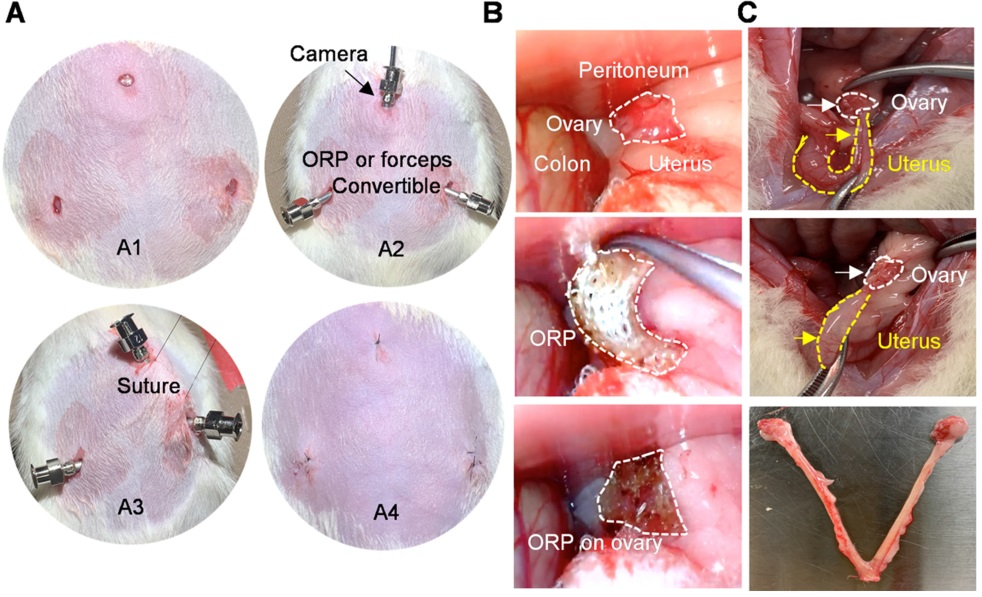
** Minimally invasive delivery of ORP. (A)** The processing of laparoscopic surgery for ORP implantation. A1) Three minimal incisions on abdomen, each less than 0.5cm. A2) Troca for micro-camera, tools and ORP. A3) Suture hanging up abdominal skin for operation space in abdominal cavity. A4) The sutures after surgery. **(B)** Laparoscopy showing ORP delivery. **(C)** Ovaries were harvested 66 days after surgery, white curve indicates ovary and yellow curve indicates uterus.

Estrus cycle stage, animal body weight, and serum reproductive hormone including estradiol (E_2_), follicle-stimulating hormone (FSH) and anti-müllerian hormone (AMH) were evaluated before and after POI induction (Figure S5). The result showed that all animals had a regular estrus cycle that lasted 4-5 days (Figure S5B, C), and their body weight, and serum E_2_, FSH, and AMH levels were not significantly different (Figure S5D). After cisplatin treatment, rats lost body weight (Figure S5D), and got different estrous cycles that were characterized by fewer proestrus and estrus events and significantly more time in metestrus and diestrus (*P* < 0.01) (Figure 3B). Additionally, cisplatin treatment decreased E_2_ and AMH, but increased FSH (Figure S5D). All these data suggest the establishment of rat POI model.

### Minimally invasive delivery of ORP to the rat ovaries

In previous studies, therapeutics for ovary were usually delivered through laparotomy 36. To mimic the clinical laparoscopy for female pelvic surgery, the rats were subjected to three minimal incisions (diameter < 0.5 cm; upper midline and the left and right lower abdomen) for synMSC injection or ORP implantation (Figure 3A). The intraabdominal photos and videos showed a practical process of minimal invasive ORP implantation (Figure 3B and Video S2). The exploratory laparotomy suggested that ORP was fully degraded 14 days after implantation (Figure 3C).

### ORP boosts retention of synMSC on the rat ovaries

**Figure 4:**
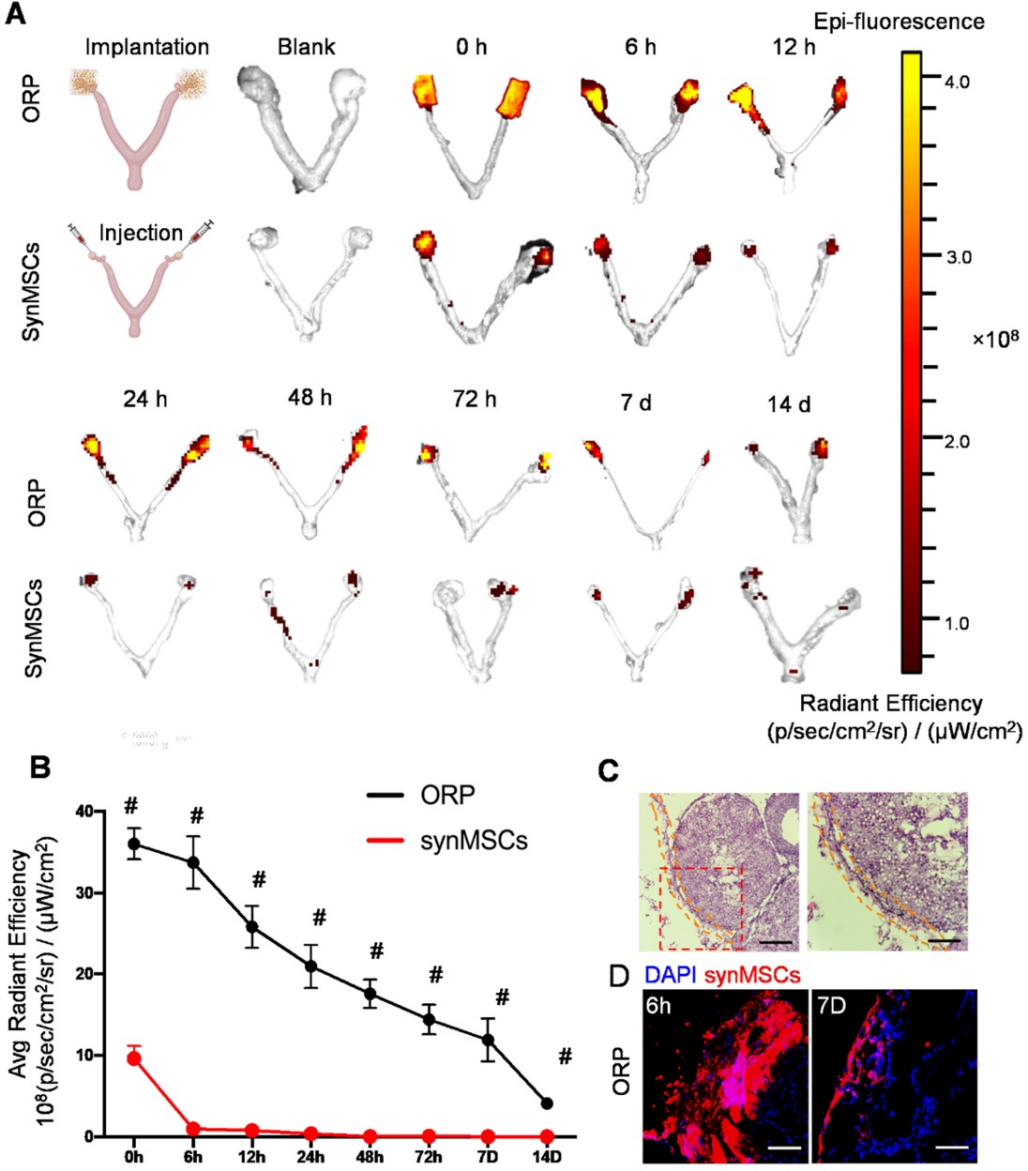
** ORP implantation boosts ovarian retention of synMSCs. (A)***In vivo* fluorescent imaging (IVIS) of POI injured ovaries at various time intervals after ORP implantation or synMSCs injection (*n* = 3). **(B)** Quantitative analysis of fluorescent intensity of ovaries (*n* = 3). **(C)** Ovary H&E staining after ORP implantation 7days, yellow curve indicates ORP, Scale bar: 500 µm, Scale bar (zoomed snapshot) = 150 µm. **(D)** Fluorescence images of synMSCs retention on ovaries 6h and 7 days after ORP implantation. Scale bar: 100 µm. All data are presented as mean ± SD. Student's t-test for comparison between two groups at same time point. All data are presented as mean ± SD. *^#^*indicates *P* < 0.0001.

To understand the advantages of ORP implantation over direct synMSC injection *in vivo*, we transplanted ORP that assembled with Rhodamine B prelabeled synMSCs via laparoscopy in rat with POI. The control animals were injected with equal amount of Rhodamine B prelabeled synMSCs. To detect the retention of synMSCs, we traced the fluorescent signals for 14 days after ORP implantation. The ovaries and uterus were harvested at indicated timepoints after ORP implantation or synMSC injection (Figure 4A and B). These results showed that the injected synMSCs were washed away as soon as the injection was performed, and fluorescence was negligible after 6 hours. In contrast, ORP implants largely retained synMSCs on the ovary. Histological studies confirmed the presence of ORP on ovaries seven days after implantation (Figure 4C). Confocal images showed increased synMSC retention in rats treated with ORP (Figure 4D). These datasets confirmed that ORP implantation increased the retention and engraftment of synMSCs.

### Biocompatibility of ORP in rats with POI

Clinical trials and evidence-based medicine have confirmed the biocompatibility and safety of 37, 38. The biocompatibility of synMSCs has been tested in our previous studies 20, 39. In this study, we tested the biocompatibility of ORP by implantation of ORPs beneath the dorsal dermis of a healthy rat with an intact immune system. Then, all the layers of skin were harvested for Hematoxylin and Eosin (H&E) (Figure S6A) and IHC analysis (Figure S6B) 7 days after implantation. The results showed that there was minimal immune cell infiltration.

### Therapeutic effects of ORP

**Figure 5:**
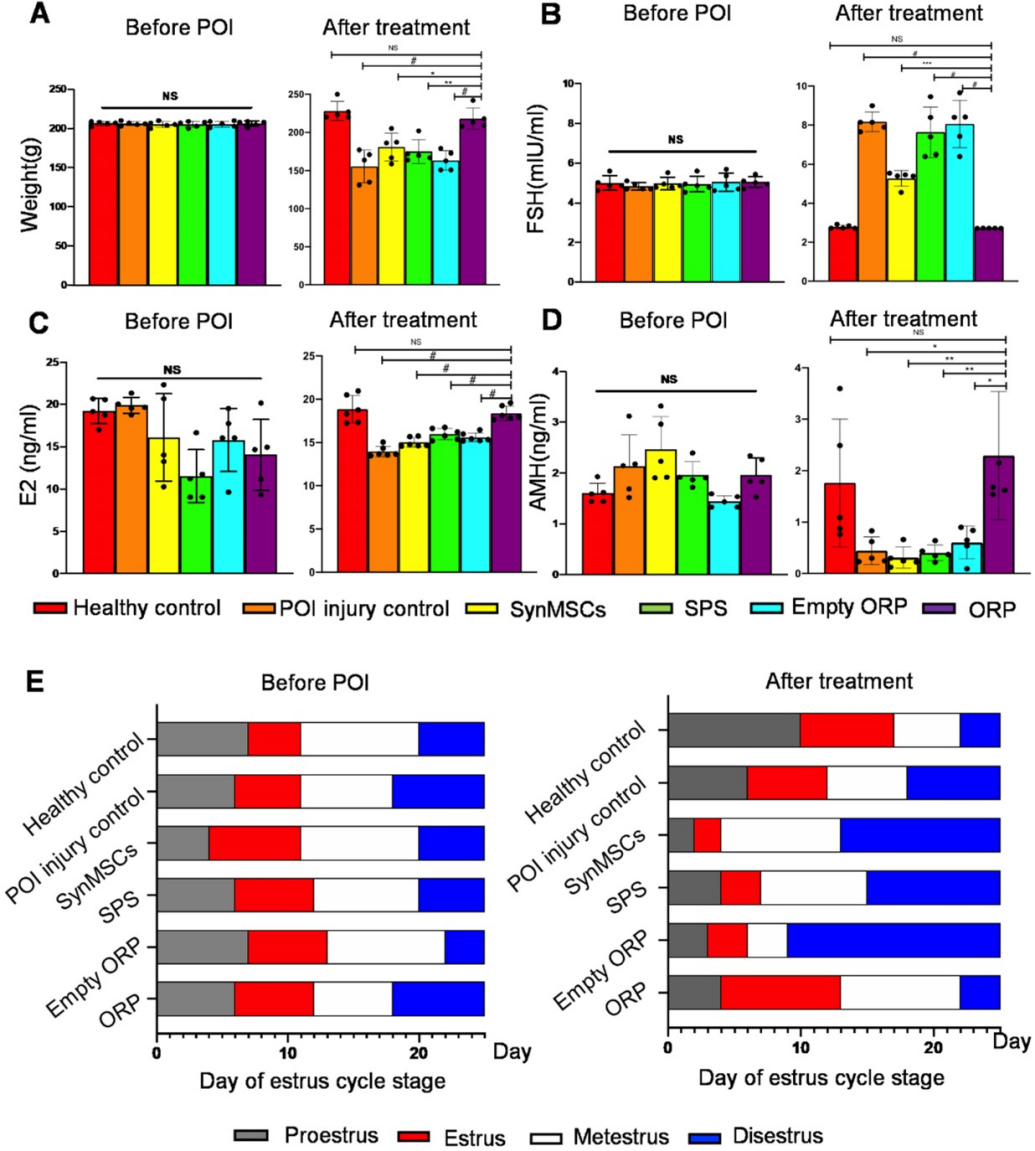
** ORP treatment restores normal hormone production and estrus cycling. (A)** Animal body weight before and after treatment (*n* = 5). Quantitative analysis of serum hormone level FSH **(B)**, E_2_ **(C)** and AMH **(D)** before and after treatment (*n* = 5). **(E)** Duration of estrous cycle stage before and after treatment (*n* = 5). All data are presented as mean ± SD. NS indicates *P* > 0.05, * indicates *P* < 0.05, ** indicates *P* < 0.01, *** indicates *P* < 0.001, and *^#^* indicates *P* < 0.0001.

To test the therapeutic effects of ORP, we randomly assigned rats into six groups (*n* = 5), including healthy controls, POI rats, and POI rats treated with synMSCs, SPS, empty ORP or ORP (Table S1). All treatments were performed 3 days after the chemical induction of POI. Estrous cycle stage, body weight, and serum reproductive hormone levels were assessed 14 days after treatment. Compared to other treatments groups, ORP treated POI rats have similar body weight, FSH, E_2_ and AMH levels compared to the healthy controls (Figure 5A-D). In addition, ORP treated rats spent less time in diestrus (Figure 5E). These results suggested that ORP implantation rescued ovarian function.

**Figure 6:**
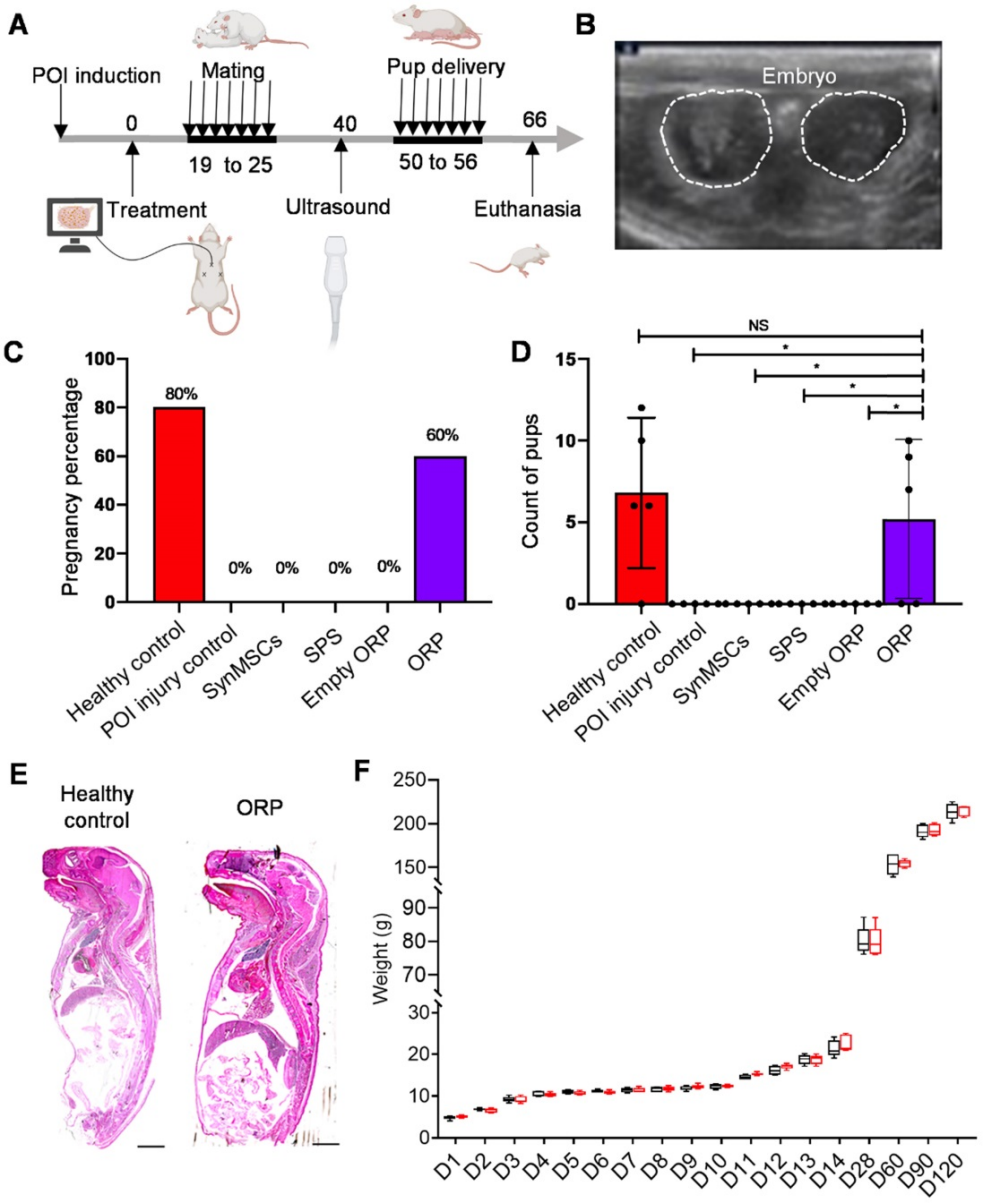
** ORP treatment rescues fertility in POI rats. (A)** Schematic showing the timeline of mating, delivery of pups and termination point after treatment. **(B)** Ultrasound image of fetus detection (via fetal heart detected) to confirm pregnancy. **(C)** The percentage of pregnancy (*n* = 5). **(D)** Count of pups after delivery (*n* = 5). **(E)** Histological image of newborn rat pups from control and ORP treated rats. Scale bar = 2mm. **(F)** Weight of offspring in healthy control and ORP from birth to day 120. All data are presented as mean ± SD. Student's t-test for comparison between two groups and one-way ANOVA for comparison among more than two groups. NS indicates *P* > 0.05, * indicates *P* < 0.05.

The outcomes of pregnancy and live birth were critical for evaluation of fertility and ovarian function 40. All groups of rats mated with healthy adult male rats for one week (Figure 6A). Two weeks later, pregnancy was confirmed by ultrasound scanning of embryo and fetal heartbeats (Figure 6B and Video S3). The result showed a rate of pregnancy for 80% and 60% in healthy control group and POI treated ORP group (Figure 6C). There was no pregnancy detected in rats treated with cisplatin alone or with synMSC, SPS or empty ORP treatment. All pups were counted at the time of delivery. The control group delivered 34 live pups (6, 12, 6, 10, respectively), while POI injured rats with ORP treatment group delivered 26 live pups (9, 7, 10, respectively). No rat got pregnant, neither pup delivered in all other groups. There is no difference between control and ORP-treated rats. However, there are significant differences between ORP and other treatment groups (Figure 6D and video S4). Additionally, no malformations or gross anomalies were detected in pups delivered by control or ORP treated mother rats. Growth curves of control and ORP treated offspring were similar in a 120-day follow-up.

**Figure 7:**
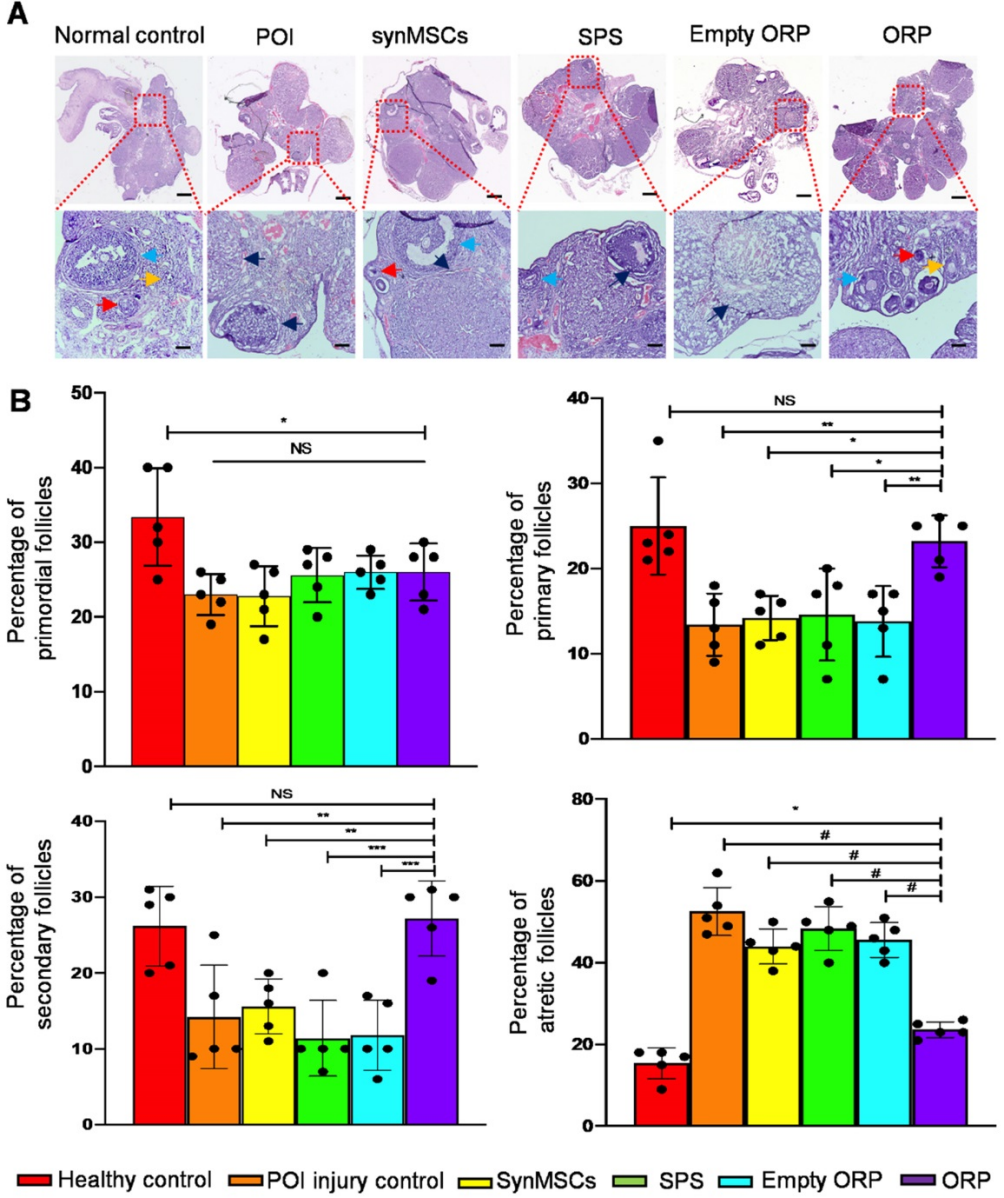
** ORP treatment increases number of primary and secondary follicles and reduces the percentage of antral follicles. (A)** Ovarian follicles in different groups 66 days after treatment. Arrows indicate primordial follicles (red arrows), primary follicles (yellow), secondary follicle (blue) and atretic follicles (black) respectively. Scale bar = 1 mm. Scale bar (zoomed snapshot) = 200 µm. (n = 5). **(B)** Quantitative analysis of primordial, primary, secondary and atretic follicles in different groups (*n* = 5). All data are means ± SD. Comparisons among groups were performed using one-way ANOVA followed by post hoc Bonferroni test. The comparisons between samples are indicated by lines, and the statistical significance is indicated by asterisks above the lines. NS indicates* P* > 0.05. * indicates* P* < 0.05, ** indicates *P* < 0.01, *** indicates *P* < 0.001, and *^#^* indicates *P* < 0.0001.

H&E staining revealed that ovaries from each group revealed follicles in different stages, such as primordial, primary, secondary and atretic follicles (Figure 7A). Rats with chemically induced POI had significantly fewer primordial follicles, indicating reduced ovarian reserve function (Figure 7B). The percentage of primary follicles and secondary follicle among total follicles in ORP treated rats was also significantly higher than rats with chemically induced POI as well as other treatment groups (Figure 7B). Moreover, the percentage of atretic follicles among total follicles in ORP treatment group was significantly lower than other cisplatin treated groups (Figure 7B). In contrast, the percentage of atretic follicles in ORP treated group was not significantly different than the healthy control group. With TEM imaging, we assessed microanatomy of ovaries of POI rats with or without synMSCs treatment. Treatment with synMSCs increased the numbers of granulosa cells and the intracellular organelles (Figure S7). Consistent with the rescue of ovarian hormone secretion, these data suggest both synMSCs and ORP attenuate the adverse effects of cisplatin on ovarian cellular constituents. Taken together, these results suggested the therapeutic effects of ORP to improved ovarian outcomes after injury.

### Potential mechanisms underlying ORP treatment in rats with POI

**Figure 8:**
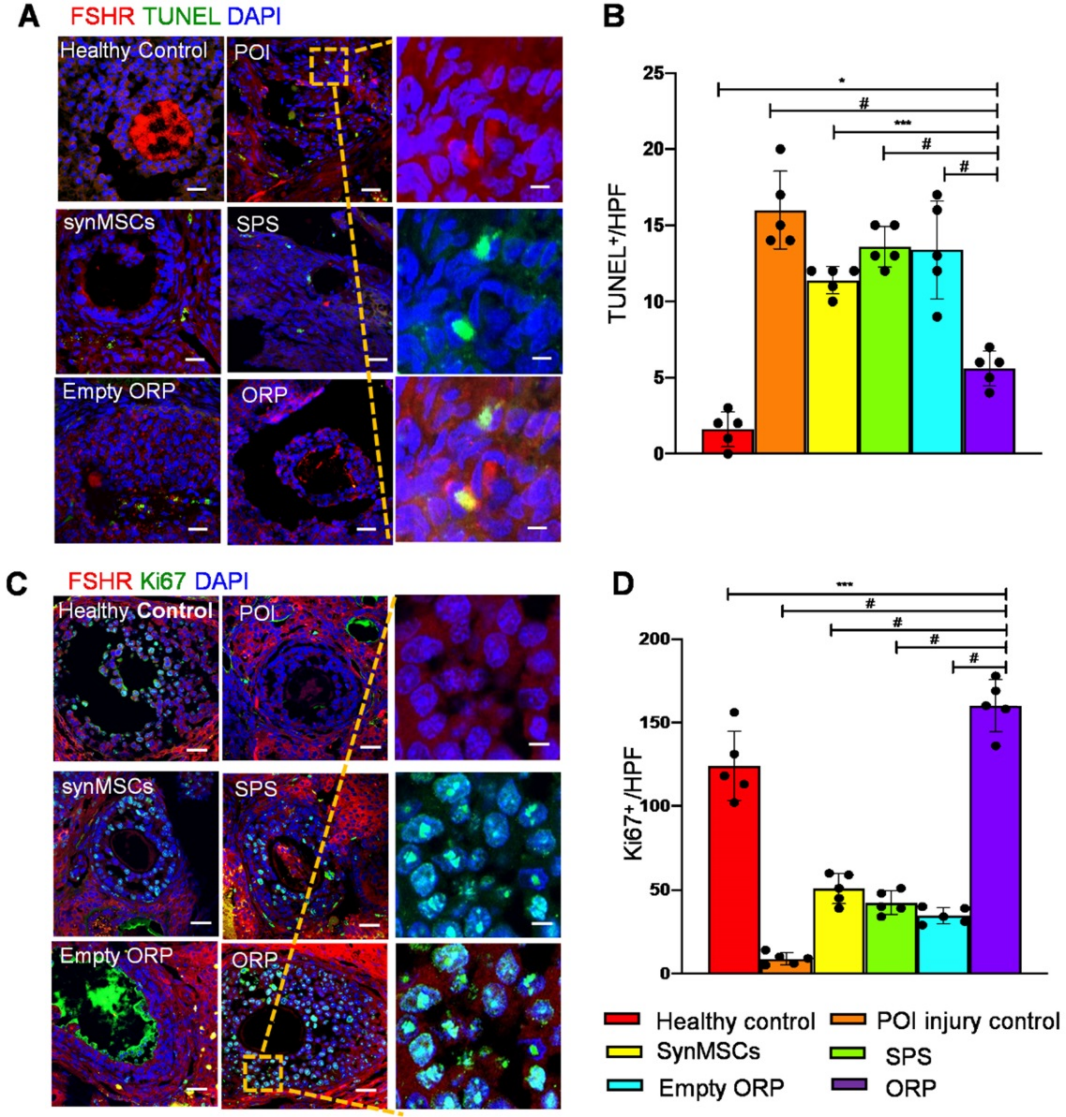
** ORP treatment reduces OSC apoptosis, and increases OSC proliferation and angiogenesis. (A)** TUNEL cell^+^ (green) was detected in control, POI injury, synMSCs, SPS, empty ORP or ORP. Scale bar: 200 µm; Scale bar (zoomed snapshot) = 40 µm. **(B)** Quantitative analysis of TUNEL^+^ cells per high-power field (HPF) (*n* = 5). **(C)** Ki67^+^ (green) cells on the follicles. Scale bars: 200 µm; Scale bars (zoomed snapshot) = 40 µm. **(D)** Quantitative analysis of Ki67^+^ cells per HPF (*n* = 5). Image J software were used for quantitative data assessment. All data are means ± SD. Comparisons among groups were performed using one-way ANOVA followed by post hoc Bonferroni test. The comparisons between samples are indicated by lines, and the statistical significance is indicated by asterisks above the lines. * indicates *P* < 0.05, *** indicates *P* < 0.001, and^ #^ indicates* P* < 0.0001.

To explore the potential mechanisms of the treatment effects of ORP, we performed Terminal-deoxynucleotidyl Transferase Mediated Nick End Labeling (TUNEL) assay to detect cell apoptosis in all groups (Figure 8A). The result indicated that ORP treatment significantly reduced apoptosis of OSCs which is detected by FSHR, compared with other cisplatin treated groups (Figure 8B). Cell proliferation was assessed via IHC of Ki67 (Figure 8C). The result showed that Ki67 expression in ovaries with ORP implantation was significantly higher than other treatment groups. These data suggest that ORP treatment reduced the adverse effects of cisplatin on proliferation of OSCs (Figure 8D). Usually, reduced stromal blood flow coincides with ovarian dysfunctional 41. Blood perfusion is essential for developing follicles and oocyte quality. To measure angiogenesis, the expression of endothelial cell marker CD31 (Figure S8A) in all animal groups were compared. ORP treatment group expressed more CD31 than other groups, which suggested that ORP enhanced angiogenesis and improved the hemoperfusion (Figure S8B). Altogether, implantation of ORP reduced OSCs apoptosis, enhanced proliferation of OSCs, and increased angiogenesis in cisplatin-induced POI.

**Figure 9:**
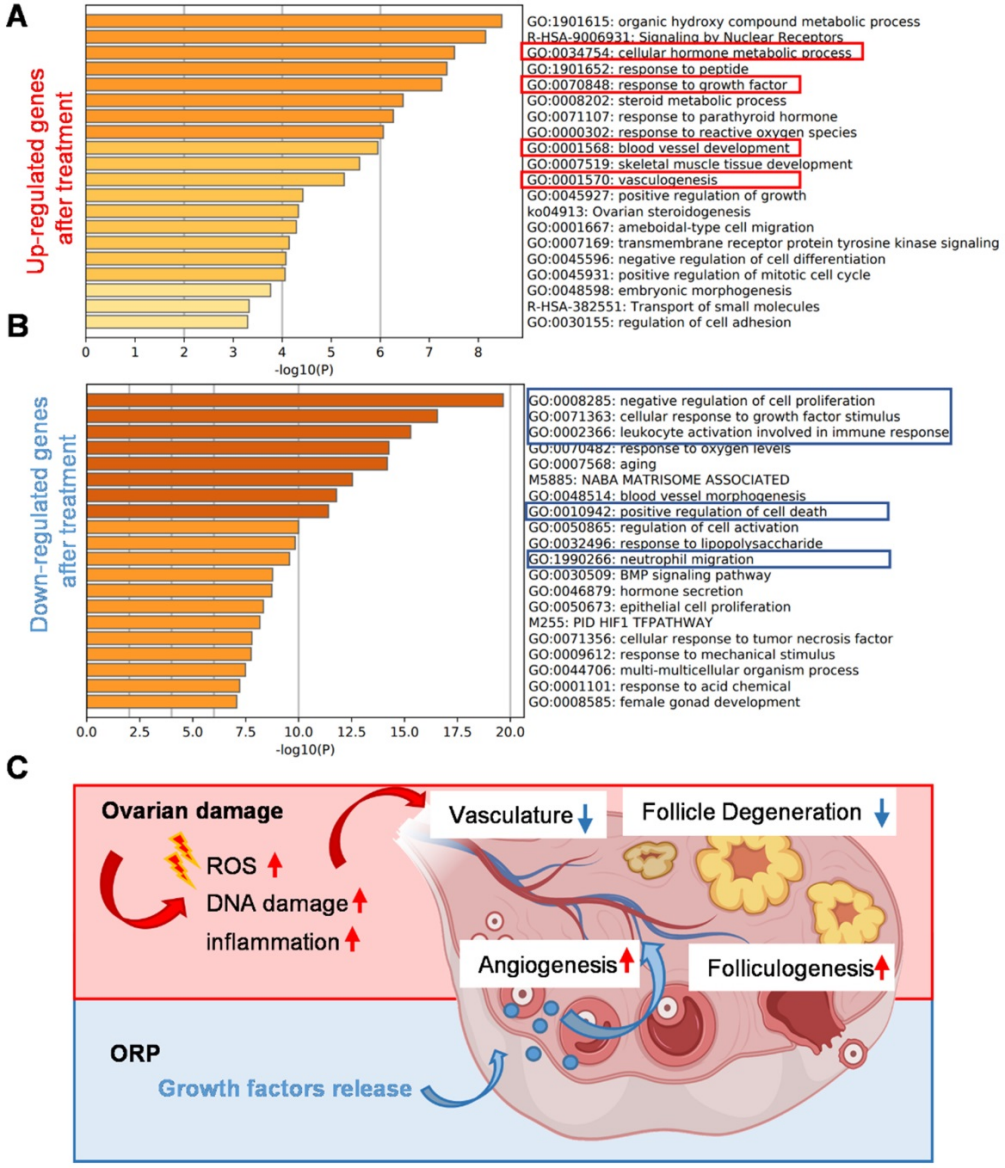
** Gene analysis and mechanism of the cisplatin-induced POI model and ORP treatment. (A, B)** Bar graph across upregulated (A) or downregulated (B) gene lists, colored by p-values. **(C)** Schematic illustration of the mechanism underlying ORP treatment.

By comparing the RNA-seq data of ovaries from the cisplatin-injured rats and ORP treated rats, we listed out the upregulated and downregulated genes after treatment and performed the Metascape Gene List Analysis according to the literature 42. As we can see from Figure 9A, ORP treatment led to an enhanced expression of genes in response to growth factors and metabolic hormone process. In addition, ORP favors the development of vascular development after the damage. In Figure 9B, we can see that ORP reduced cisplatin-induced upregulation of genes that adversely affect free radical formation and inflammation.

## Discussion

Over the past decades, stem cell therapies have been proposed as a promising treatment for POI patients 41, 43. Huc-MSC has been reported effective for POI treatment via restoration of ovarian function 44. However, direct implantation of live cells may increase the risk of developing carcinoma and immune reactions. Stem cell-derived secretome has been studied widely as an alternative treatment that restored serum level of FSH and E_2_ in rats with iatrogenic POI 13 and augmented fertility in advanced reproductive aged animals 12. The clinical application of these secretomes is still hampered by the issue of *in vivo* retention and sustainable release 45, 46.

Patching up the tissues represented a sustained delivery manner for therapeutics 47. In this study, we fabricated a fully acellular ORP by embeding synMSC particles in a widely used SPS. New approach of ORP adopts a “particle in matrix” depending on the proven helpful effects of synMSCs and SPS. SynMSCs achieved their function via mimicking the paracrine process of live MSCs 23. GDF-9 can influence the differentiation of all follicle compartments, such as oocytes, granulosa cells and theca cells. The ovaries from newborn GDF-9 knockout mice, isolated oocytes cannot recruit the surrounded OSCs to form follicles 48*.* TGF-β signaling pathway contributes multiple functions for the mammal ovaries. When inhibin and activin were knocked out, TGF-β family ligands, secreted from OSCs, caused an increasing number of ovarian function deficiency because of interference in the regulation between pituitary and ovaries. In both murine and humans, AMH, a TGF-β family ligand, is an ovarian growth factor, can regulate the recruitment of primordial follicles and the sensitivity of FSH in follicle development 49. VEGF has been demonstrated to enable for arteriogenesis and improve development of granulosa cells by increasing the vessels of length, area, and branches 50. HGF can inhibit apoptosis on OSCs and oocytes to improve vascular area growth, which contributes to ovarian function 51. The SPS scaffold was a widely used material for reducing clinical relevant consequences of local wound bleeding and tissue adhesions 26.

ORP treatment protected ovarian function by rescue of female sex hormones, estrous cycling, follicle development and fertility. We observed increased numbers of ovarian follicles when we delivered ORP in cisplatin-treated POI rats. However, it is unclear if ORP treatment rescued or prevented oocyte and granulosa cells from cell death or if there was regeneration of ovarian follicles subsequent to cisplatin-induced POI. However, a healthy woman can ovulate 400-500 oocytes in her life span, fully developed and ovulated follicles is less than 1% in total primordial follicles 22. So, therapies to improve oocyte quality and follicle development are the key to solve the problem.

The rat model of chemically induced POI is a limitation of this work. Pilot studies in a nonhuman primate may offer more information about the utility of ORP in humans. To understand the molecular biological mechanisms by which ORP therapies attenuate the adverse effects of cisplatin on ovarian reserve and function. Additionally, the duration of Huc-MSC secretome releasing and ORP degradation need to be optimized *in vivo*. Nevertheless, our present study presents a highly effective and clinically feasible new therapeutic strategy for POI.

## Supplementary Material

Supplementary video S1.

Supplementary video S2.

Supplementary video S3.

Supplementary video S4.

## Abbreviations

AMH: anti-müllerian hormone
DCM: dichloromethane
FSH: follicle-stimulating hormone
FSHR: Follicle-stimulating hormone receptor
FITC: fluorescein isothiocyanate isomer
FBS: fetal bovine serum
GDF-9: growth differentiation factor-9
HRT: hormone replacement therapy
Huc-MSCs: human umbilical cord mesenchymal stem cells
E_2_: estradiol
HGF: hepatocyte growth factor
IACUC: Institutional Animal Care and Use Committee
ORP: ovarian regenerative patch
OSCs: ovarian somatic cells
PLGA: polylactic-co-glycolic acid
PBS: phosphorylated buffer saline
POI: Primary ovarian insufficiency
PVA: poly (vinyl alcohol)
SPS: surgical pelvic scaffold
synMSCs: synthetic mesenchymal stem cells
SEM: scanning electron microscopy
TUNEL: Terminal-deoxynucleotidyl Transferase Mediated Nick End Labeling
TGF-β: transforming growth factor-β
VEGF: vascular endothelial growth factor
